# Direct evidence of a low barrier hydrogen bond in the catalytic triad of a Serine protease

**DOI:** 10.1038/s41598-018-28441-7

**Published:** 2018-07-04

**Authors:** Peter Agback, Tatiana Agback

**Affiliations:** 1 Department of Molecular Sciences, Swedish University of Agricultural Sciences, PO Box 7015, SE-750 07 Uppsala, Sweden; 2 A&A Structure and Dynamics, SE-754 71 Uppsala, Sweden

## Abstract

Serine proteases are one of the largest groups of enzymes, found in both eukaryotes and prokaryotes, and are responsible for many different functions. The detailed information about the hydrogen-bonds in the catalytic triad (Asp…His…Ser) of these enzymes is of importance in order to fully understand the mechanism of action. The aspartate of the triad is hydrogen bonded to the histidine but the exact nature of this bond has been under discussion for some time. It is either a common short ionic hydrogen bond (SIHB) or a delocalized low barrier hydrogen bond (LBHB) were the hydrogen bond is shorter. So far, the evidence for LBHB in proteins have not been conclusive. Here we show clear NMR evidence that LBHB does exist in NS3, a serine protease from Dengue. The one bond coupling constant between the hydrogen and nitrogen was shown to be only 52 Hz instead of the usual 90 Hz. This together with a ^1^H chemical shift of 19.93 ppm is evidence that the hydrogen bond distance between His and Asp is shorter than for SIHB. Our result clearly shows the existence of LBHB and will help in understanding the mechanism of the catalytic triad in the important group of serine proteases.

## Introduction

The incidence of dengue virus (DENV) has grown around the world in recent decades and constitutes a major threat to human health. With increased infection rates, aided by global warming, the growth of urban areas, travel and trade, the virus is now endemic in more than 100 countries and not only restricted to tropical and subtropical regions thus making it an important subject for detailed studies.

**Figure 1:**
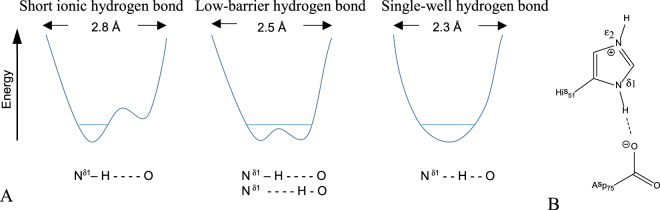
(**A)** Energy profiles for the possible hydrogen bonds for His…Asp. (**B)** The hydrogen bond between HNδ1 of His51 and Asp75.

NS3 of Dengue type II (DENV2) virus is a serine protease and belongs to the large and functionally diverse group of proteolytic enzymes. Serine proteases continue to be an inspiration for mechanistic studies of enzyme catalysis and intensive efforts have been made to address questions regarding the mechanism of the serine protease catalysis^1,2^. One of the common features of serine proteases is the catalytic active site which consist of the triad of aspartic acid, histidine, and serine residues, Asp…His…Ser, in an interactive mode. Still, the nature of the hydrogen bond (HB) stabilization in the catalytic triad is the subject of discussion. There remains the question whether the Asp…His hydrogen bond in the catalytic triad is associated with a normal short ionic hydrogen bond (SIHB)^3,4^ or with a delocalized resonance effect called a low-barrier hydrogen bond (LBHB) or with a single well hydrogen bond (Fig. 1)^5–7^. Theories, such as the LBHB hypothesis, are inherently difficult to prove experimentally in biomolecules due to the very short-lived nature of their transition states. Furthermore, these controversies also arise from inadequate characterization of H-bonds in proteins.

The type of hydrogen bond is of importance to our understanding of the structure of the tetrahedral transition state and the possible intermediates which are necessary for a functioning enzyme. There are multiple experimental demonstrations of the transition state stabilization by HBs where a tetrahedral intermediate has been observed by a series of intermediate trapping experiments^8,9^. The LBHB proponents and opponents have shown several experimental observations which could be categorized into the following groups: (a) unusual low-field chemical shift (CS) of protons involved in H-bonding^3,5,6,8^ (b) measurement of H-bonding length by ultra-x-ray^10^ (c) PK studies using ^15^N and ^13^C nucleus^11^ (d) H/D isotope effect studies^12^.

Characteristic shifts in NMR chemical shielding are a well-known signature of H-bonding. The primary focus of most of the studies has been on the NMR chemical shielding effects of the catalytic His aromatic nuclei; ^1^H, ^15^N and ^13^C. Unusual large low- field shift of the N^δ1^H protons, for single-well H-bonds (20–22 ppm) and for LBHB (17–19 ppm) has been proposed as one of the main criteria for the formation of a short, strong hydrogen bond under protonation of the catalytic His in complex with Asp in the intermediate induced complexes of serine type proteases with inhibitors^5^. This has been confronted in many theoretical^4,13^ and experimental studies^3,14^. It was argued that the large down field shifts that is seen for the proton between His….Asp^4^ is equally consistent with both the LBHB and the SIHB^15^. Diffraction experiments carried out using X-rays or neutrons give a direct image of the atomic positions, including that of the hydrogen atoms, however, hydrogen atoms are only visible when crystal diffraction data are at ultra-high resolution (i.e. better than 1 Å) and where the atoms are sufficiently well ordered^10^. The coordinate uncertainty should be much less than 0.1 Å in order for a reliable detection of geometric changes needed to distinguish between different types of H-bonds^10^.

Scalar through-H- bond J-coupling ^1^*J*_AH_, ^2^*J*_AHB_ in A-H···B have been shown to provide an important window on H-bonding by directly exhibiting the intermolecular electronic delocalisation and ‘communication’ that underlies H-bond formation^16–18^. There has been shown a direct correlation between ^1,2^*J*-coupling, the low field chemical shift of proton and the length of the H-bond in organic molecules and DNA^17,19,20^. Unfortunately, experimental values of ^1^*J*-coupling are often not available for many H-bonded system of biological interest. This means that the currently applied experimental diagnostic of H-bonding criteria in the catalytic triad is generally incomplete and missing the direct experimental evidence of the one bond ^1^*J*_NH_ coupling constant from NMR data. Furthermore, those few available NMR studies where the ^1^*J*-coupling in the catalytic Asp…His was observed demonstrated that all the measured values of the one bond ^1^*J*_NH_ lay between 87 and 95 Hz and that the proton was localized on N^δ1^H for at least 85%^21^.This fact was used as convincing evidence against the LBHB theory^2^ where much smaller ^1^*J*_NH_ coupling are predicted^14^.

The virally encoded serine protease of Dengue virus serotype, type 2 (DENV2) lies in the N-terminal domain of NS3, with NS2B serving as a cofactor in this dimeric protease. Full length NS2B is a membrane protein, but *in vitro* a hydrophilic segment of 40-residues is sufficient to form an active NS2B-NS3pro complex^22^.

In the presence of ligand, the DENV NS2B C-terminus associates with NS3pro such that it lines the active site. This NS2B conformation is referred to as the ‘closed’ conformation, and is distinctly different from that observed for ligand free protease structures^23–25^. The C-terminal part of NS2B has been shown essential for proteolytic activity in both DENV^26^, WNV^27,28^ and Zika^29^ therefore the closed conformation is thought to be the enzymatically active structure.

The X-ray structure obtained so far of the active conformation of the DENV3 NS2B-NS3pro complexes (67% sequence identity with DENV2) with inhibitor have not had enough resolution to measure hydrogen bonds^30^. A low resolution modelled structure based on NMR data was recently reported, again it is not possible to say anything about the possible presence of LBHB^31^. The x-ray structures from related viruses are also lacking the resolution necessary to estimate the H-bond distance between His and Asp in the catalytic triad^27–29^. It is also not clear if the structures obtained from all these x-ray investigations shows an active conformation in which one would expect to observe LBHB. An important suggestion from the study of West Nile virus NS3 is the proposal that the active site of NS3 undergoes an induced fit involving the substrate and histidine^28^. This could explain the lack of LBHB in all apo forms of NS3.

## Results and Discussion

**Figure 2:**
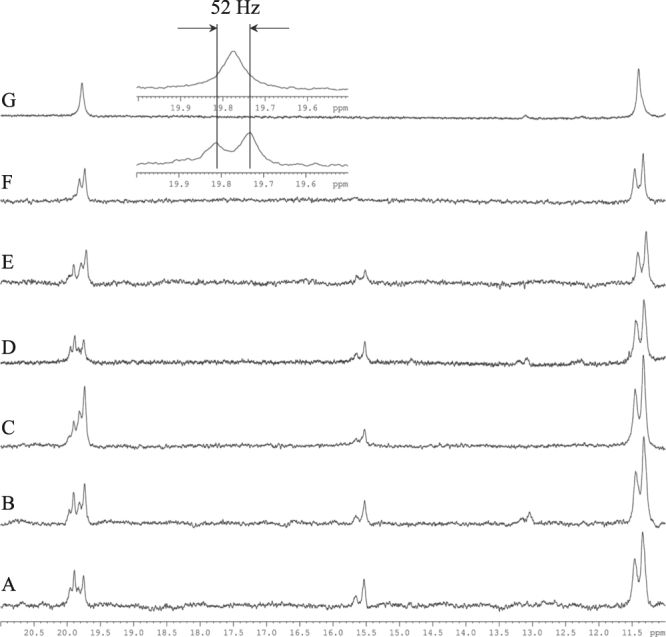
1D ^1^H spectra of the region 11–21ppm of the complex ^15^N/^13^C labelled NS3-NS2Bpro with, Bz-Nle-Lys-Arg-Arg-B(OH)_2_, without ^15^N/^13^C decoupling at different pH: **(A)** 5.5, **(B)** 6.0, **(C)** 6.5, **(D)** 7.2, **(E)** 8.5 all in MES buffer. In **(F)** pH 8.5 in Tris buffer and **(G)** unlabeled NS3 in the same complex at pH 8.5 in Tris buffer. The small insert shows N^δ1^H of His51 at 19.772 ppm with the one bond J-coupling ^1^J_Nδ1H_ = 52 Hz indicated.

Here we present experimental NMR evidence, which to the best of our knowledge, is the first time that the existence of a LBHB type H-bond for the Asp…His interaction is found in the complex of DENV2 with a substrate-analogue boronic acid mimicking the tetrahedral transition complex. Binding of the Bz-Nle-Lys-Arg-Arg-B(OH)_2_, to the NS3:NS2Bpro complex leads to significant changes in the NMR spectra of the complex^32^. In Fig. 2 ^1^H low field spectra with or without ^15^N and/or ^13^C decoupling of the complex ^15^N/^13^C-NS3:NS2Bpro with Bz-Nle-Lys-Arg-Arg-B(OH)_2_ with pH varying between 5.5–8.5 are presented. There are four signals observed between 20–19 ppm and two signals around 15.57 ppm. One should note that in the absence of the ligand, no resonances are observed between 20 and 13 ppm. The pH dependent studies of the complex were performed in order to investigate any changes in pKa coming from changes in the protonation of the histidine^18,33,34^. A histidine not hydrogen bonded is expected to show chemical shift dependence on pH whereas histidine bound in a complex do not^18,33,34^. All three sets of signals (doublets in Fig. 2) in the 20–14 ppm region do not show any pH-dependence on their chemical shift and thus remain fully protonated and H-bonded over the investigated pH range.

When decoupling of ^15^N and/or ^13^C nuclei is applied or when only the co-factor NS2B was uniformly ^15^N/^13^C labelled, the resonances are reduced to two signals at 19.933 ppm (J_NH_ coupling = 52 Hz), 19.772 ppm (J_NH_ coupling = 52 Hz) and one at 15.57 ppm (J_NH_ coupling = 90 Hz) (Figs 2, S1 and S2).

This means that those signals belong to one of the aromatic NH residues of NS3pro. A spectra of the mutation of His51Asn of NS3pro in complex with NS2B and the boronic ligand does not show any extreme low field signals at 19.933, 19.772 and 15.57 ppm thus confirming the assignment of His51, see Fig. S3 in supplementary materials.

**Figure 3:**
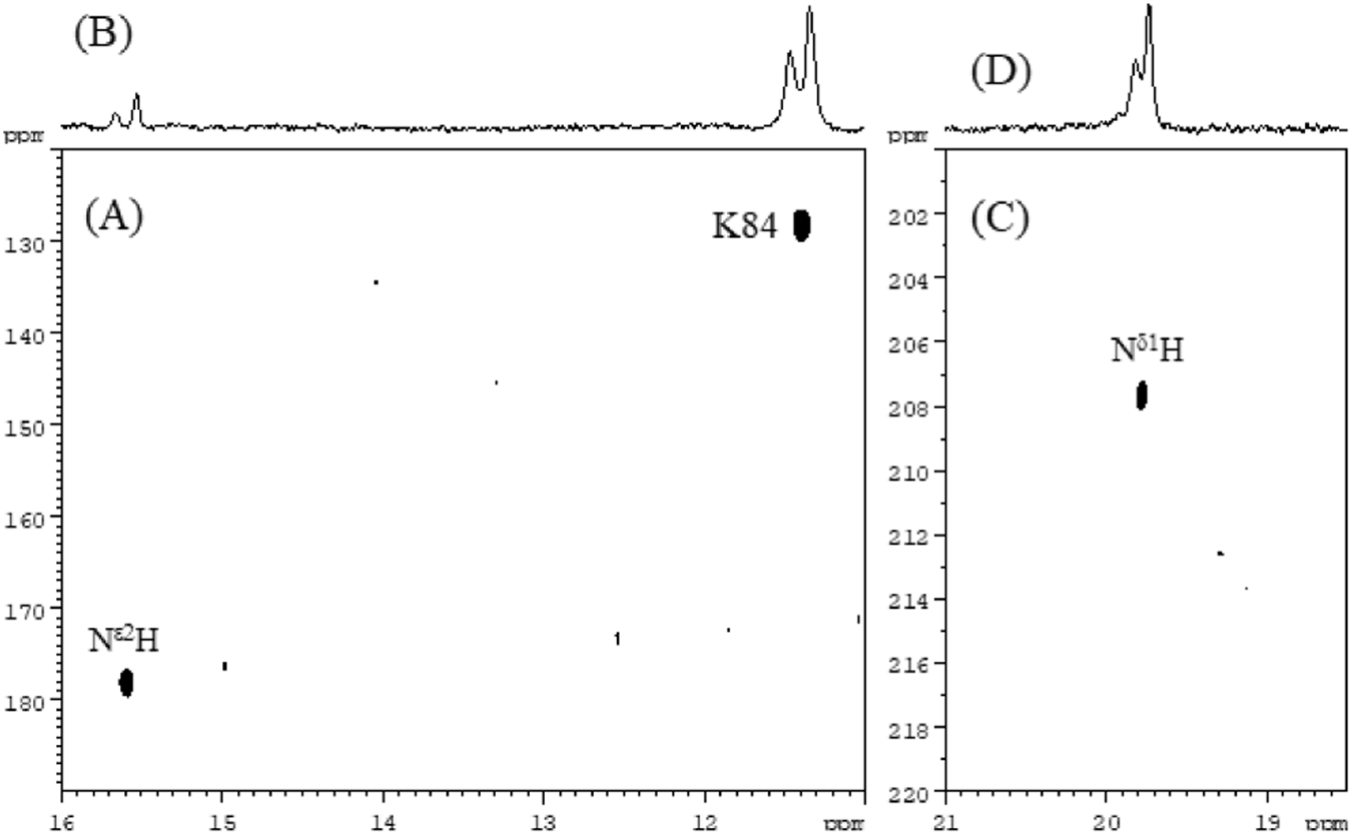
Expanded 2D plots **(A)** and **(C)** of ^1^H-^15^N HSQC TROSY type spectra of the complex ^5^N/^13^C labelled NS3-NS2Bpro with Bz-Nle-Lys-Arg-Arg-B(OH)_2_, showing one bond ^1^H-^15^N correlation with corresponding 1D ^1^H spectrum presented on top **(B)** and **(D)** of the 2D spectra. In expansion **(A)** amide cross peak of amino acid K84 at 11.396/127.85ppm is shown as reference. Cross peak at 15.587/178.7ppm is assigned to the N^ε2^H of His51. In expansion **(C)** cross peak at 19.772/207.6ppm is assigned to the N^δ1^H of His51 of the other form.

For the signal at 19.772 ppm we were able to detect a one bond ^1^H and ^15^N correlation cross peak (^15^N: 207.9ppm) in the TROSY type ^1^H ^15^N HSQC spectrum (Fig. 3) which allows us to unambiguously assign it to N^δ1^H of His 51. For the signal at 15.57ppm ^1^H and ^15^N correlation cross peak also detected with ^15^N: 178.7ppm (Fig. 3) which assigned to N^ε2^H of His 51. The above assignment is based on the x-ray structures of serine proteases showing a possible hydrogen bonding between N^δ1^ of a histidine and Asp. For the other resonance at 19.933ppm we failed to observe any ^1^H and ^15^N cross peak possibly due to different NH coupling constant (52 Hz instead of 90 Hz), line broadening and the lower concentration of the second form of the complex.

Note that there seems to be an equilibrium between two different types of complexes of the catalytic triad depending on external conditions (Fig. 2). One form, with the most intense signals at 19.772, is the most persistent in all spectra at different buffers and pH. The other one has signals at 19.933 and 15.57. This will be discussed more below.

To rule out the contribution of the ^2^*J*_C-H_ scalar coupling to the observable splitting we have also examined ^15^N-NS3:NS2Bpro with Bz-Nle-Lys-Arg-Arg-B(OH)_2_ complex: still the coupling constant of the signals at 19.933 ppm and 19.772 ppm were 52 Hz. The coupling is about 38 Hz less than commonly reported for catalytic HN His in serine proteases which claimed to be in range 87–95Hz^14^. Another striking feature is that signals at 19.933 ppm and 19.772 ppm assigned by us to N^δ1^H protons of the His51 in the catalytic triad are observed at almost 1.0 ppm more downfield than so far reported for either N^ε2^H or N^δ1^H protons in protonated His induced by inhibitors^9,14^. However, the observed chemical shifts and one-bond coupling constants are the expected ones in LBHB if one extrapolates the results of a study of chemical shifts and coupling constants in the catalytic triad of NS3 in flaviviruses^23^. Only in the complex with trifluoromethyl ketone has a downfield shift at 18.9 ppm been observed and this was the main reason to claim the presence of the LBHB^5^. The ratio of the integrals of those three signals varies with different pH: at pH 6.5 the ratio was 0.25:0.5:0.25 correspondingly, but at pH 5.5 and 7.2 it was close to 0.3:0.3:0.3. As mentioned above, it indicates that there are an equilibrium between different forms of binding of the boronic type of inhibitor. Resonances at 19.933p pm and 15.57 ppm belong to the His of the same complex. Remarkably, the resonance at 15.57 ppm assigned to N^ε2^H proton and 19.933 ppm to belong to the same protonated His 51 but have different ^1^*J*_NH_ couplings of 90 Hz and 52 Hz, respectively. Furthermore, depending on the sample preparation, one or more complex forms could be observed. As an example, in Tris buffer at pH 8.5 only one down field signal is observed, at 19.772 ppm, but the two other ones are reduced below the detection limit (Fig. 2). The difference between the two complex forms is not clear to us yet but they both show the same ^1^*J*_NH_ coupling of N^δ1^H of 52 Hz and thus LBHB.

The reason that we don’t think that the observed effects are solely linked to the presence of boron are the following: boronic type ligands are regularly used in the studies of serine proteases in order to mimic the intermediate complex. No unusual structural and functional features of the complexes have been reported in these studies^35–37^. The hydrogens of the His in the catalytical triad are more down field than usual in these complexes, but not to extent reported by us, and the coupling constants are either ca 90 Hz or not reported. This indicates that the presence of the boron does not in itself induce any major conformational change. Subsequently we do not think that the boronic ligand is the main reason for our down field shifts and coupling constant. Instead we believe that the reason is that we managed to trap a true mimic of the otherwise short living intermediate of the active complex due to the presence of the NS2B co-factor.

In conclusion from the above presented results it becomes clear that the small ^1^*J*_NH_ coupling of 52 Hz of N^δ1^H of His 51 indicates that the proton in the HB is located almost equidistant between Asp75 and His51 in the complex of NS3:NS2B:ligand. This result is the first evidence that serine proteases, at least for Dengue Type II, exhibit LBHB or possibly even single-well HB between the catalytic aspartate and histidine in the active site as proposed by Frey *et al*.^5,38^. Without the ligand or in free NS3, we have not observed any evidence of hydrogen bonding between His51 and Asp75. This is consistent with the induced fit proposal in the study of West Nile virus NS3 mentioned above. Our result obtained for the investigated construct indicates that we here have a very close mimic to the real transition-state of an active enzyme. Our reported findings will, together with more structural work on the complex NS3:NS2B:ligand, facilitate the development of rational structure based inhibitors that can selectively target the NS3 protease of Dengue type II (DENV2) virus. Further studies of the structure and dynamics of the complex presented here, including the two forms of His51, are ongoing.

## Methods

### Protein expression and purification

Reagents were from Sigma (St. Louis, MO, USA) unless otherwise stated. DENV2 NS3pro (1–185; amino acids 1476–1660 of the polyprotein) and NS2B (containing amino acids 1394–1440 of the Dengue 2 polyprotein) constructs were generated as described^32^. Proteins were expressed in Terrific Broth medium (MP Biomedicals) for unlabelled protein or in different isotopic labelling combinations in ^1/2^H, ^15^N, ^12/13^C-labelled M9 medium for labelled protein. In this study we mainly used ^15^N/^13^C labelled NS3 and unlabelled NS2B to form the complex as we wanted to focus on the catalytic triad and maximize the amount information. His51Asn mutation of the active site residue of NS3pro was introduced using the QuikChange Lightning kit (Agilent). All sequences were confirmed by Sanger sequencing.

Chemicals for isotope labelling (ammonium chloride, ^15^N (99%), D-glucose, ^13^C (99%), deuterium oxide) were purchased from Cambridge Isotope Laboratories, Inc.

#### Purification

NS2B and NS3pro were co-refolded by one-step dialysis overnight at 4 °C in a 2:1 molar NS2B:NS3pro ratio to maximize formation of the active complex. The refolding buffer was 25 mM Tris pH 8.5 (pH set at 4 °C), 5% glycerol, 100 mM NaCl. Thrombin (GE Healthcare) and/or TEV protease was added to a dialysis cassette (3,500 or 7,000 MWCO Slide-A-Lyzer, Thermo Fisher Scientific) to cleave off the His tag from NS2B and/or NS3pro. After refolding the solution was centrifuged at 50,000 × g to remove any precipitate or particles. Refolding yield was determined by measuring protein concentration of the two IMAC pools (NS2B: ε 5,500, MW 7.7 kDa; NS3pro: ε 36,400, MW 21.0 kDa) before refolding and comparing that to the protein concentration after refolding and centrifugation (complex: ε 41,940, MW 28.7 kDa), using a Nanodrop 1000 instrument (Thermo Scientific). The complex was then purified on an ÄKTA Explorer (GE Healthcare) by size exclusion on a HiLoad Superdex 200 column (GE Healthcare) in SEC buffer: 50 mM Tris pH 8.5 (4 °C), 5% glycerol, 50 mM NaCl.

### Protease inhibitors

The NS3pro inhibitors Bz-Nle-Lys-Arg-Arg-B(OH)_2_ used in this study was synthesized according to the reaction schemes published in the original paper^32^.

### Preparation of NMR samples

The NS2B-NS3pro complex was concentrated in disposable centrifugal concentrators (e.g. Amicon Ultra centifugal filter units) with a molecular weight cut-off of 10 kDa. The complex was stable during concentration and no leakage of NS2B occurred. Buffer was exchanged using gravity flow desalting columns (GE Healthcare). The MES NMR buffer contained 20 mM deuterated MES, 100 mM NaCl, 5 mM CaCl_2_, 0.02% NaN_3_, at pH 5.5, 6.0, 6.5, 7.2 or 8.5. The Tris NMR buffer contained 20 mM deuterated Tris, 100 mM NaCl, 5 mM CaCl_2_, 0.02% NaN_3_, at pH 8.5. The buffer-exchanged protein was concentrated to at least 0.3 mM.

Complexes of trifluoromethyl ketone peptidic inhibitor with other type of serine proteases have shown a downfield 1 H shift at 18.9 ppm and that has been used as the main reason to claim the presence of LBHB^5^ in the catalytic triad. We have observed similar behavior in Dengue NS3pro:NS2B complex with the same inhibitor (data not shown)^32^. To confirm that the boronic peptidic inhibitor used in this study binds in the same binding site as trifluoromethyl ketone peptidic inhibitor we used the following procedure.

The ternary NS2B-NS3pro-inhibitor complex was prepared in two steps. Firstly, the inhibitor 2,6-di-fluoro-Bz-Nle-Lys-Arg-Arg-CF_3_-ketone was titrated to a final concentration of 1 mM by adding 3 μL to the NS2B-NS3pro sample from a 100 mM stock solution in D_2_O. The formation of bound complex was monitored by the reduction of the ^19^F signal of unbound inhibitor and appearance of the broad signal corresponding to bound inhibitor. Secondly, the Bz-Nle-Lys-Arg-Arg-B(OH)_2_ inhibitor was added to the sample to replace the Bz-Nle-Lys-Arg-Arg-CF_3_-ketone inhibitor, which was monitored over time until the ^19^F signal representing the bound inhibitor had disappeared and the ^19^F signal of unbound inhibitor had fully reappeared.

### NMR spectroscopy

NMR experiments were acquired on Bruker Avance III spectrometers operating at 14.1 and 16.4 T at a temperature of 298 K. Backbone assignment was performed as described previously^32^. The 1D ^1^H experiments were performed using the standard Bruker parameters for zgesp with a sweep width of 35 ppm. For one bond correlation 2D ^1^H-^15^N TROSY experiments (trosyetf3gpsi.2) have been used again with standard parameters and extended sweep widths: 35 ppm for ^1^H and 160 ppm for ^15^N. ^1^H chemical shifts are in ppm downfield from DSS. ^15^N chemical shifts are in ppm vs standard reference, liquid NH_3_.

## Electronic supplementary material

Supplementary materials
