# Supplementary material for: Direct evidence of a low barrier hydrogen bond in the catalytic triad of a Serine protease
Source: Sci Rep. 2018 Jul 4;8:10078. doi: 10.1038/s41598-018-28441-7 (PMC6031666; doi:10.1038/s41598-018-28441-7)
Supplement: Supplementary file 1 — Supplementary materials [file 41598_2018_28441_MOESM1_ESM.pdf]

Direct evidence of a low barrier hydrogen bond in the catalytic triad of a Serine protease

Authors: Peter Agback[1*], Tatiana Agback[2]

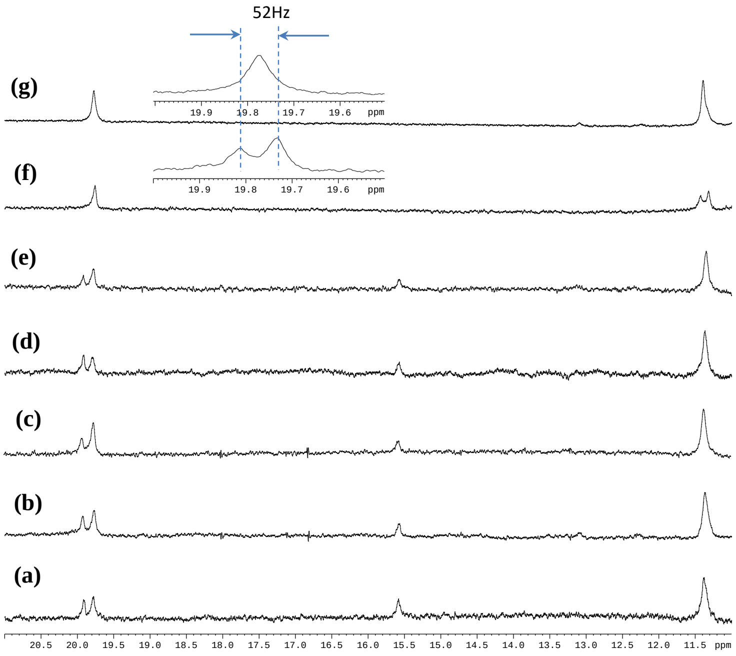

Fig S1. 1D ^{1}H spectra of the region 11-21ppm of the complex $^{15}N/^{13}C$ labelled NS3-NS2Bpro with, Bz-Nle-Lys-Arg-Arg-B$(OH)_2$, with $^{15}N/^{13}C$ decoupling at different pH: (A) 5.5, (B) 6.0, (C) 6.5, (D) 7.2, (E) 8.5 all in MES buffer. In (F) pH 8.5 in Tris buffer and (G) unlabelled NS3 in the same complex at pH 8.5 in Tris buffer. The small insert shows $N^{\delta1}H$ of His51 at 19.772 ppm with the one bond J-coupling $^{1}J_{N\delta1H}$ =52Hz indicated.

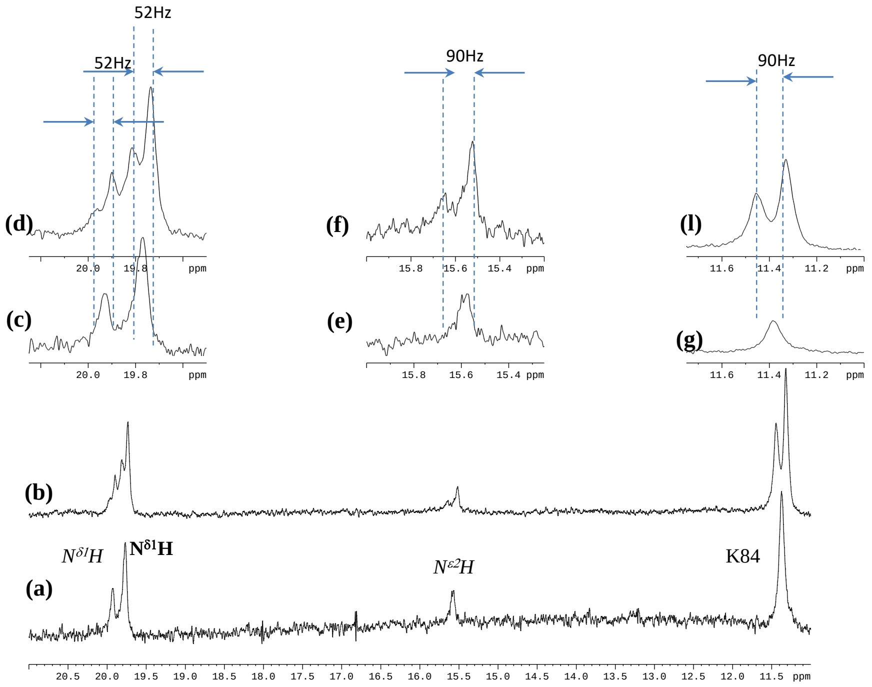

Fig S2. 1D ^{1}H spectrum between 21-11ppm of the complex ^{15}N/^{13}C labelled NS3-NS2Bpro with, Bz-Nle-Lys-Arg-Arg-B(OH)$_2$, with (a) and without (b) ^{15}N/^{13}C decoupling. In extensions resonances with coupling constants, $^{1}J_{N\delta 1H}$ =52+/-2Hz for 19.933ppm of $N^{\delta 1}H$ of His51 of the first form (in *italic*) (c),(d); $^{1}J_{N\delta 1H}$ =52+/-2Hz for 19.772ppm of $N^{\delta 1}H$ of His51 of the second form (in **bold**) (c),(d); $^{1}J_{N\varepsilon 2H}$ = 90+/-2Hz for 15.587ppm of $N^{\varepsilon 2}H$ of His51 of the first form (in *italic*) (e),(f); and as reference amide backbone $^{1}J_{NH}$ = 90+/-2Hz of the amino acid K84 at 11.396ppm (g),(l) is shown.

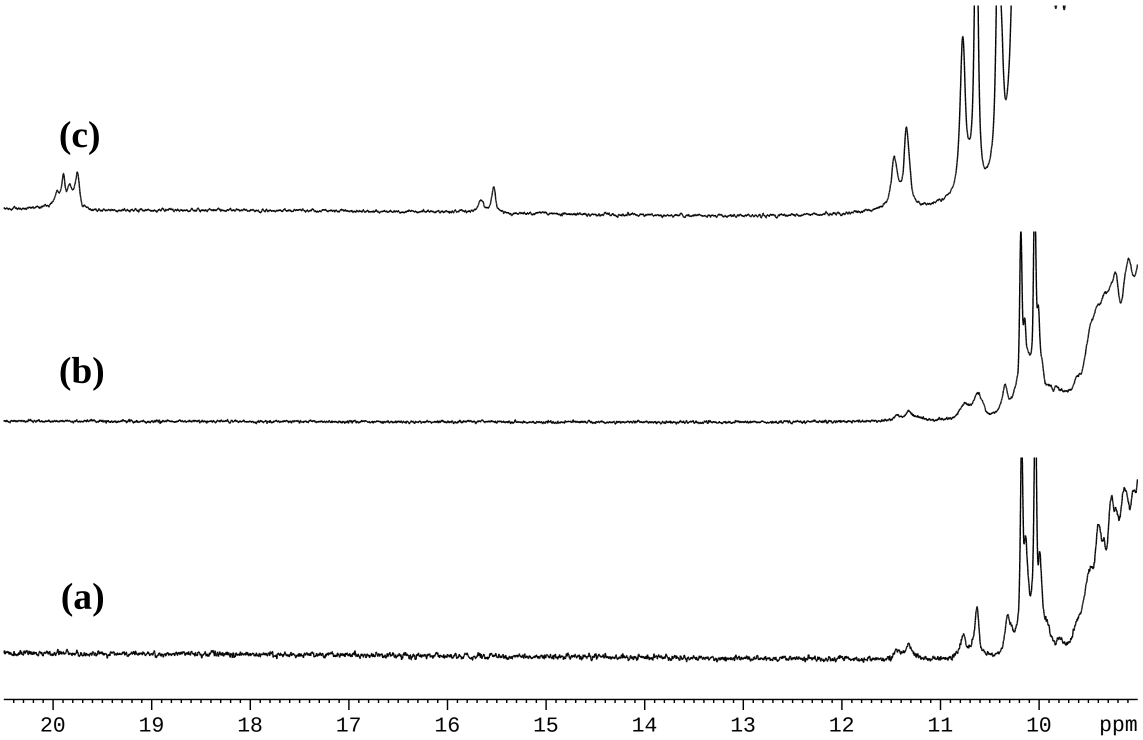

Fig S3. 1D ^{1}H spectrum in the range of 20.5-9ppm of the complexes of (a) apo $^{15}N/^{13}C$ labelled NS3-NS2Bpro; (b) H51N mutant $^{15}N/^{13}C$ labelled NS3-NS2Bpro with, Bz-Nle-Lys-Arg-Arg-$B(OH)_2$ (c) $^{15}N/^{13}C$ labelled NS3-NS2Bpro with, Bz-Nle-Lys-Arg-Arg-$B(OH)_2$.
